# Progress in Otology

**Published:** 1901-09-14

**Authors:** 


### PROGRESS IN OTOLOGY.

THE local anæsthetisation of the external auditory meatus by cocaine has hitherto been well nigh impracticable, because the cul-de-sac of the meatus, being a dermic and not a mucous surface, is virtually insusceptible to aqueous solutions of the drug. The tympanic cavity is capable of being rendered insensitive, but great caution is requisite in presence of large perforations of the drum membrane, through the liability to toxic symptoms owing to absorption by way of the Eustachian tube. Thanks to the researches of Gray, of Glasgow, great facilities can now be enjoyed in the local application of cocaine, not only to the meatus and intact membrane, but also in nose and throat work. In his first paper Gray[1] published the result of many painstaking observations made with a solution of cocaine in rectified spirit and aniline; more recently he has widened the limits of applicability of this combination, and given details of a plan for largely increasing the penetrating power of cocaine, without corresponding risk of intoxication. Two solutions are made, (1) a 20 per cent. solution of cocaine hydrochlorate in rectified spirit; (2) a 15 to 20 per cent. solution of $\beta$-eucaine in aniline oil; this does not all dissolve, so before use the bottle containing it is well shaken up, and 10 measured minims are poured out. To this are added 10 minims of No. 1 solution, and the mixture becomes clear in a few seconds, giving the formula:—Cocaine and $\beta$-eucaine, of each 10 parts; aniline and rectified spirit, of each 50 parts. These solutions are best kept apart till just before use, as the mixture otherwise soon turns brown, and perhaps loses a little of its highly penetrating quality. For ear work, the solution may be poured into the meatus while the head is turned to one side, or a small strip of gauze may be soaked in it, and pushed down the meatus to the required depth.

The list of insects and parasites recorded as having visited or infested the external auditory meatus in this country is very limited. There are one or two authentic instances of earwigs entering it, and the writer of this review once extracted a house cricket from the ear of a child upon whose drum membrane it had fastened, causing much fright and pain; also the common house flies have been known to deposit maggots. In foreign countries a more extensive fauna creates more scope in the study of human parasites infesting the ear, and Christian Simpson[3] has added a fact to our knowledge concerning the ticks or *ixodidæ*, and described an interesting case. A patient, seen in England, but who had camped out in Arizona, somehow became the host of a species of tick known as *Argas megnini*. There had been originally one in each ear canal, but that in the right side had crawled out the day previous to seeing the patient. Simpson removed the tick from the left ear, where it was embedded in wax, and found no injury inflicted upon either drum. There was a peculiarity about this tick which an expert, Mr. E. G. Wheler, considered new; on the dorsal aspect of the hinder extremity were two perforated cone-like projections through which a pointed organ could be rapidly protruded or withdrawn.

Circumscribed furunculosis of the external meatus is an acute or subacute inflammatory affection, running in the former case a short but extremely painful course. It has a peculiar tendency to periodical recurrence, owing probably to some constitutional fault. It may also set up postaural œdema simulating periostitis of the mastoid, the auricle being displaced downwards and forwards as in that disease. To explain this, as emphasised by Connal,[4] who describes a case, one has to remember the deficiencies in the cartilaginous framework of the meatus—the transverse fissures of Santorini, and the longitudinal gap in the roof. These are occupied by fibrous tissue continuous with the loose cellular tissue over the mastoid process and around the ear respectively. Hence septic inflammatory material is capable of spreading by continuity from one to the other. Œdema from the same cause may extend forwards and affect the eyelids of the same side.

Diffuse external otitis commonly occurs as a complication of acute or chronic middle-ear suppuration, or, being the primary affection, middle ear disease may result from it, and not only so, but polypi may occur, and disease of the bone in the walls of the meatus. One of the consequences of this aggravated form is atresia of the meatus. For this intractable condition a post-aural operation may have to be performed, but unfortunately there is a constant tendency to contract up again during the course of the healing process. The operation, however, allows of a thorough exploration of the fundus of the meatus, and cavity of the middle ear. In some cases the meatus will be found to be full of granulation tissue springing from necrosed bone; whilst on the surface of granulations, may develop a cuticular envelope, forming a complete or perforated diaphragm at the orifice of the meatus. MacNaughton Jones[5] exhibited a patient to the Otological Society in whom the meatus was completely closed, following scarlet fever and measles. She had a quantity of dense tissue removed by operation, but in the course of healing contraction again took place, and a silver-gilt tube was inserted to keep the parts open. A temporary discarding of the tube, owing to irritation, resulted in complete re-growth of tissue and closure of the orifice. The encroaching material was once more removed with the galvano-cautery knife, and the tube re-inserted; the latter is now worn permanently. The alternative to this course would be the complete radical operation, a very wide opening being made and skin grafting resorted to.

[1] Lancet, Ap. 21, 1900. [2] Ibid., Mar. 9, 1901. [3] Ibid., Ap. 27, 1901. [4] Brit. Med. Jour., May 25, 1901. [5] Transactions Otol. Soc., Vol. I., p. 61.

